# How patient and community involvement in diabetes research influences health outcomes: A realist review

**DOI:** 10.1111/hex.12935

**Published:** 2019-07-08

**Authors:** Janet Harris, Johannes Haltbakk, Trisha Dunning, Gunhild Austrheim, Marit Kirkevold, Maxine Johnson, Marit Graue

**Affiliations:** ^1^ School of Health and Related Research (ScHARR) University of Sheffield Sheffield UK; ^2^ Faculty of Health and Social Sciences Western Norway University of Applied Sciences Bergen Norway; ^3^ Centre for Quality and Patient Safety Research Deakin University and Barwon Health Partnership Geelong Victoria Australia; ^4^ Library Western Norway University of Applied Sciences Bergen Norway; ^5^ Department of Nursing Science, Institute of Health and Society University of Oslo Oslo Norway

**Keywords:** community engagement, diabetes, participatory research, partnership working, patient involvement, realist review

## Abstract

**Background:**

Patient and public involvement in diabetes research is an international requirement, but little is known about the relationship between the process of involvement and health outcomes.

**Objective:**

This realist review identifies who benefits from different types of involvement across different contexts and circumstances. Search strategies Medline, CINAHL and EMBASE were searched to identify interventions using targeted, embedded or collaborative involvement to reduce risk and promote self‐management of diabetes. People at risk/with diabetes, providers and community organizations with an interest in addressing diabetes were included. There were no limitations on date, language or study type.

**Data extraction and synthesis:**

Data were extracted from 29 projects using elements from involvement frameworks. A conceptual analysis of involvement types was used to complete the synthesis.

**Main results:**

Projects used targeted (4), embedded (8) and collaborative (17) involvement. Productive interaction facilitated over a sufficient period of time enabled people to set priorities for research. Partnerships that committed to collaboration increased awareness of diabetes risk and mobilized people to co‐design and co‐deliver diabetes interventions. Cultural adaptation increased relevance and acceptance of the intervention because they trusted local delivery approaches. Local implementation produced high levels of recruitment and retention, which project teams associated with achieving diabetes health outcomes.

**Discussion and Conclusions:**

Achieving understanding of community context, developing trusting relationships across sectors and developing productive partnerships were prerequisites for designing research that was feasible and locally relevant. The proportion of diabetes studies incorporating these elements is surprisingly low. Barriers to resourcing partnerships need to be systematically addressed.

## 1 INTRODUCTION

Research on the social determinants of health shows that the living conditions of people with diabetes, including cultural background, economic circumstances and built environment, can interfere with the potential effectiveness of interventions.^1^ Culturally adapting interventions can increase their relevance, acceptability and uptake of physical activity and healthy eating.^2^ Adapting an intervention refers to ‘the process of altering a program to reduce mismatches between its characteristics and those of the new context in which it is to be implemented or used’^3^ (p.25).

If mismatches between intervention and target group are reduced, the reach and provision of services may become more equitable for people in minority or vulnerable groups.^4^

A recent meta‐review found 199 reviews published since 2010 on patient and community involvement in adapting interventions.^4^ However, there are no published studies concerning the effectiveness of involving patients and the public in adapting diabetes interventions to reduce diabetes risk or achieve better diabetes self‐management.^5^

### 1.1 The theory of involvement in research

People can be involved in many different types of research. The focus of our review was involvement in co‐designing and implementing interventions to help people reduce diabetes risk and self‐manage diabetes. The underlying theory for involvement posits that people who have a health condition, their carers and their communities have an important contribution to make in terms of identifying and prioritizing research topics, as well as the ways the research should be conducted.

Involvement enhances research in a number of ways as follows: ensuring the relevance and appropriateness of the research design; developing more effective recruitment strategies; designing research tools that are more appropriate and user‐friendly; conducting interviews and surveys with more relevant and acceptable lines of enquiry; analysing data that includes lay perspectives; and better dissemination and implementation of research findings.^6^

The lived experience of people with chronic health conditions and their carers is just as important as the professional knowledge of practitioners and the skills of health researchers,^7^, ^8^ so contributions at any of these steps could influence the relative success of an intervention. Research can be done *to* a community, *for* a community, *by* a community or *with* a community. Although these different stances reflect different perspectives on involvement, the relationships between stance and effectiveness are rarely reviewed.^9^

Although involvement is stipulated by an increasing number of funders and organizations, we need to know more about how patient involvement can actually contribute and in what circumstances it is useful.^10^ The International Diabetes Federation (IDF) Guidelines recommend that people with diabetes work with organizations to provide expert support and recommend that lay health workers actively engage people in communities, responding flexibly to dimensions of culture, ethnicity, psychosocial situations and disability.^11^ Such engagement, which is conceptualized as a ‘meeting of minds coming together’,^12^ is based on the principle that the experiences of people who have diabetes and those at risk of diabetes are essential in the collaborative design and delivery of services that work for everyone.

Despite the IDF Guidelines, diabetes research has been challenged by issues such as low uptake of screening and problems with recruitment to education sessions.^13^, ^14^ Diabetes research has also been criticized for not considering context, erring on the side of focusing on interventions to change individual behaviour, when effectiveness is actually influenced by factors outside an individual's control,^15^ including physical environment (access to healthy affordable food, opportunities for activity), psychosocial context (exposure to stressors, mental health and coping strategies) and biological characteristics.^16^ A recent review suggests that culturally tailored diabetes interventions may increase participation by minority and migrant groups.^17^

Despite the evidence that co‐produced health research can ultimately improve service delivery, patient experience and outcomes,^18^, ^19^ we did not identify any reviews exploring the relationships between various types of involvement and diabetes outcomes.

We conducted a review to identify how different approaches to involvement are being used to design and adapt diabetes interventions, and whether involvement contributes to reduction of diabetes risk and improved self‐management.

The questions for the review were as follows:

RQ 1: How have people with diabetes and the wider community been involved in setting priorities, designing and conducting diabetes research?
RQ 2.1: What are the main characteristics of the process that appear to explain the relative success or failure of involving people with diabetes and the wider community in diabetes research?
RQ 2.2: Does successful involvement in adapting diabetes interventions benefit people with diabetes, communities and practitioners, leading to achievement of health outcomes?

## 2 METHODS

Our questions reflect a contingent review design (Table 1) where findings from the first set of included projects inform the retrieval and synthesis of subsequent projects to answer different questions within the review.^20^ A two‐stage approach to identifying literature was used because we aimed to do a realist synthesis, and our realist synthesis was dependent on identifying projects that describe the process of participation.

**Table 1:** Overview of the contingent review design

Review question aims	Projects included if they	Output
RQ1: Scoping and mapping to identify projects that involve people with diabetes and the wider community	Report actual involvement at one or more stages of the project	Identification of type of involvement by stage
RQ2.1: Identifying characteristics contributing to success or failure of involvement	Discuss reasons for success or failure of involvement	Propositional statements illustrating how interactions in different circumstances promote or preclude involvement
RQ2.2: Establishing whether there are relationships between the type and level of involvement and achievement of health outcomes	Discuss or establish relationships between the process of involvement and outcomes	A mid‐range theory explaining how involvement can work at different stages of the research project in patients with diabetes and the wider community to promote achievement of positive health outcomes

Realist synthesis was used to explore how involvement was facilitated or hindered by different contexts (RQ2.1) and whether interactions between researchers, patients and communities influenced the design, delivery and outcomes of the intervention.^21^

We chose a realist approach because, although the degree of involvement can be seen and reported, the underlying explanations for involvement or processes that trigger involvement are hidden.^22^ Involvement may be influenced by a number of aspects of the surrounding context. For example, researchers may be reluctant to involve people because they cannot see the value of it, they may feel that the time needed cannot justify the expense, and they may feel unequipped with the requisite skills for engagement.

People with diabetes may lack confidence to participate because they do not understand research or recognize the value of their lived experience. These attitudes and feelings about engagement act as mechanisms, which either enable or constrain involvement in research. These mechanisms have been documented in reviews of patient involvement.^6^ However, the ways in which contexts act to either create negative attitudes and feelings towards diabetes interventions, or mitigate and promote positive mechanisms for managing diabetes, have not been systematically documented. Realist synthesis enables the interactions between contexts and mechanisms to be mapped and related to outcomes.^23^ It is therefore a promising approach to explaining what works for whom and in what circumstances to promote diabetes involvement.

### 2.1 Scoping and mapping

A sensitive search was conducted (GA, JH) in order to determine how many diabetes research studies actively involved patients and the wider community in diabetes intervention studies. The scoping was used to make decisions on how to organize studies, based on their respective contexts, populations and approaches to involvement.

The scoping search strategy was based on previous systematic reviews of patient and community involvement in research.^6^, ^24^ We searched health databases (Medline, CINAHL, EMBASE), and search terms were broad in order to map the extent of involvement. Population was defined as people with diabetes or at risk for diabetes who are patients, potential patients, carers of people with diabetes and people from organizations that represent people who use diabetes services. The wider community was defined as: health and community service providers, community organizations with an interest in addressing diabetes and diabetes risk, researchers and policymakers.

Involvement was defined as engagement, collaboration or partnership working across the population and wider community that aimed to co‐design and co‐implement interventions that drew upon the expert knowledge of all those who were involved. Involvement in diabetes research was defined as participation in the priority setting, design, implementation and/or evaluation of diabetes initiatives.

All interventions reducing risk for developing diabetes and promoting self‐management of diabetes were included. There were no limitations on date or language. All study types were included, as diabetes interventions have used a range of designs from clinical trials to community‐based participatory research.^4^

**Figure 1:**
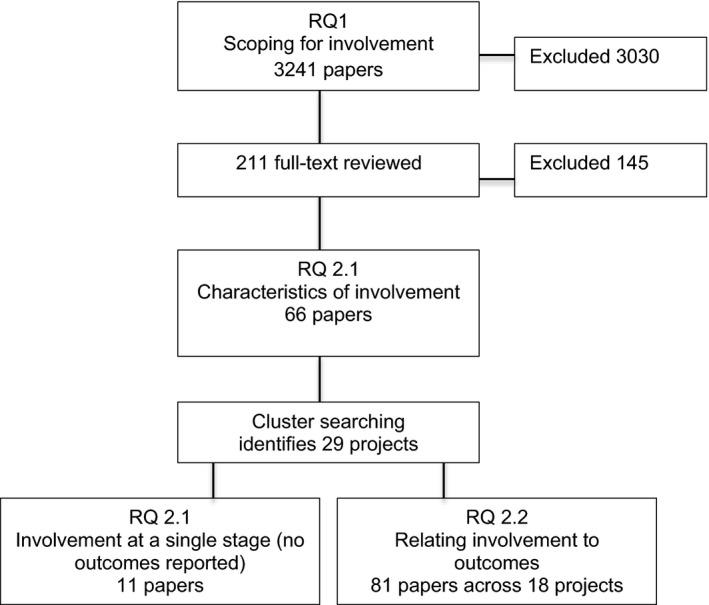
Flow chart for study selection at each stage of the review

As projects were identified, we identified a number of issues concerning completeness of reporting involvement. We attempted to address these ‘thin descriptions’ by locating all of the papers for each project using a technique called ‘cluster searching’ (see Figure 1). Project names and members of the author team were used to identify additional papers describing a particular project.^25^

Several of the projects noted that they were aiming to pilot or conduct trials measuring diabetes outcomes, so the search was updated in 2016 and again in 2017 to identify the completed trials and also checked for additional projects. We concurrently searched for papers that presented theories or conceptual frameworks for involvement. Several typologies and frameworks were identified that have conceptualized involvement in health research.^26^, ^29^, ^30^, ^31^, ^32^

The scoping search that was conducted in 2015 produced 3241 hits. After removing duplicates and papers that were not about diabetes, 2716 titles and abstracts were screened for relevance by dividing the set across the review team (JH, MG, MK, JHa, TD). Relevance was defined as the pertinence of the paper to the review question and concordance with the inclusion criteria.^27^ A total of 3030 abstracts were excluded, primarily because involvement was not defined. Undefined involvement referred to a research study where there was no public involvement in the planning, design or conduct of the research, or where the claim of involvement was not explained or evaluated.^26^

In a number of cases, it was necessary to review the full text because information on involvement was not clear in the abstract. A further 145 papers were excluded because (a) some claiming involvement did not actually report on it; and (b) some incorrectly described retrospective interviews or focus groups conducted after the study to explain what happened as involvement.

We used a recent conceptual analysis of involvement 26 to classify projects by types of involvement (see Box for definitions).

Box 1 **Types of involvement^26^**

1

- Targeted consultation: people are contacted and consulted on specific aspects of the study, for example tasks such as a research proposal, wording of information sheets or surveys. Those involved may not be otherwise involved in the design of the study and may not receive much information regarding subsequent progress, outputs or impact.
- Embedded consultation: People with relevant lived experience are consulted regularly throughout the research cycle from initial ideas and proposals to dissemination of findings. People may be individual representatives on steering or advisory groups; or be representing a user‐led organization. The research team retains ownership and control over the research study with regular input from the public.
- Collaboration and co‐production: People with relevant lived experience are active members of the research team, contributing to key decisions regarding the research process as well as the findings. Relationships are reciprocal and collaborative, with shared control across researchers, patients and the public, based on specific areas of expertise.
- User‐led research: People with lived experience are supported to lead the research, and take a systematic approach to directing the team through each stage from selecting the topic, writing proposals, designing the intervention, collecting and analysing data, and disseminating findings.

As the primary aim of most of the papers was to report on a diabetes intervention rather than on involvement, papers needed to be assessed for relevance.^28^ Descriptions of involvement were scattered across the introduction, background and discussion sections of the paper in many instances. The quality of information within the papers was assessed on ability to contribute:

- data and insight about the contribution of involvement to the process of designing and implementing diabetes interventions;
- explanations of how involvement may be related to diabetes outcomes.

The final set totalled 92 articles (see Reference section for detailed list of papers by project).

Data extraction was done by members of the team (JH, MG, JHal, MK) with a subset of articles, in order to develop a data extraction template based on previously developed frameworks.^29^, ^30^, ^31^, ^32^ The first data extraction template was structured by the stage of the project at which participation occurred: priority setting, proposal writing, intervention design and implementation. Implementation included involvement in recruitment, delivering the intervention, data collection and analysis. We then used a recently published concept analysis for community involvement to conduct the synthesis.^26^

There were four concepts contained in the framework that were reported in projects, which we used to develop the theory of involvement. These were as follows:

- Frequency of contact, for example one‐off or specific consultation vs regular contact.
- Contact vs collaboration: People who interact for a specific purpose have a different relationship with the researchers than people who actively collaborate as members of the research team.
- Reciprocal relationships are developed when people with relevant lived experience are able to contribute their knowledge and skills as equal members of the team.
- Shared control over the research process leads to identification of relevant topics and production of knowledge that is useful for diabetes management.

Propositional statements describing the relationship between context, mechanisms and outcomes were drafted for each project (JH). Other members of the team critiqued the statements for coherence (JHal, MG, MK, TD). The statements were subsequently refined and patterns (demi‐regularities) were identified between the types of involvement used in different contexts and outcomes of involvement.

## 3 RESULTS

Projects were the unit of analysis for this review, rather than individual articles, so results are presented by citing projects (see Reference section).

RQ 1: How have people with diabetes and the wider community been involved in setting priorities, designing and conducting diabetes research?

The 29 projects were situated in six countries (UK, USA, Australia, Canada, New Zealand, Ireland) and covered a range of ethnic groups (Maori, Aborigine, Native American, North American, Latino, Asian, African American, Caribbean, British, Irish). Eight focused on adults (Brown 2006, Carlson 2006, Evans 2007, Gadsby 2012, Lee 2007, Lindenmeyer 2007, Paul 2007; Simmons 2013), with the remainder focusing on families and community residents. Projects that focused on the wider community included people at risk of diabetes as well as those who were already diagnosed (Thompson 2000; Braun 2002; Daniel 1999; Goldfinger 2008; Adams 2004; Mendenhall 2010; Macaulay 1997; Merriam 2009; Coppell 2009; Hanley 1995). Four projects were conducted in clinical settings (Noyes 2014; Paul 2007; Simmons 2013; Peek 2008); one initiative expanded to include a range of community settings (Peek 2008). The remaining projects were community‐based, initiated by academic‐community partnerships.

The projects were organized by types of involvement (see Box 1). Targeted and embedded involvement took place in a context where researchers organized one‐off or limited consultation with patients or members of the public to inform a specific research element (Table 2).

**Table 2:** Targeted and embedded involvement

Author, Year, Country	Involvement approach	Contextual drivers for research	Contribution of involvement					
			Priority setting	Study design	Study information & recruitment	Educational materials	Data collection analysis	Dissemination
Brown, 2006, UK	Focus groups	NHS policy to involve consumers, but there is a lack of research on involving people from deprived areas and minority ethnic groups	Y	U	N	NA	Y	NA
Gadsby, 2012, UK	Partnership Voting	Interests of clinicians, patients and carers may be overlooked when priorities are set by funding bodies and academics	Y	U	U	NA	U	U
Lee, 2007, Australia	Focus groups Partnership	Need to tailor consumer medicine Information via consumer involvement	U	U	U	Y	U	U
Paul, 2007; Smith, 2011, UK	Researcher controlled trial Semi‐structured interviews	Peer support may be a more effective approach to involving patients in self‐managing diabetes than didactic support	N	Y	N	N	N	N
Simmons, 2013; 2015, UK	Researcher controlled trial Semi‐structured interviews Observation	Although peer support can be effective in diabetes, little is known about differences in effectiveness between individual and group peer support	N	Y	N	N	N	N
Noyes, 2010; 2014, UK	Interviews, focus groups	Lack of child‐centred research has hampered development of effective interventions	U	Y	N	Y	N	U
Evans, 2007, UK	Tailoring educational toolkit Action research	Uncertainty regarding management of pre‐diabetes in primary care	U	U	U	Y	Y	U
Lindenmyer, 2007, UK	Research user group Qualitative case study	Need to ascertain what makes user involvement successful in a research user group that has been established for 6 y	Y	Y	Y	Y	Y	U
Mudd‐Martin, 2013, US	Partnership CBPR	Need to reduce risk of type 2 diabetes among genetically susceptible	Y	Y	Y	Y	Y	Y
Schoen 2010, Australia	Community reference group Focus groups	No freely available diabetes foot care information for Aboriginal population	U	Y	Y	Y	Y	Y
Thompson, 2000, Australia	Community‐based ethnography Survey pilot	Knowledge of cultural risk factors is not integrated into epidemiological risk factor surveys	U	Y	Y	Y	Y	Y
Watson, 2001, Australia	Partnership CBPR	Culturally appropriate tools for diabetic foot care needed	Y	U	U	Y	U	Y

Abbreviations: N, Did not contribute; NA, Not Applicable; U, Unknown; Y, Contributed.

Two projects focusing on priority setting had frequent contact and one‐off contact, respectively^46^^,^ 43. Gadsby et al^46^ established a partnership representing diabetes organizations, diabetes research networks, people with diabetes and carers. The partnership set up a steering group that had eight face‐to‐face and teleconference discussions prior to the priority setting exercise. A democratic process produced an agreed set of the priorities that were subsequently taken to funding bodies. Brown et al^43^ used primary care staff to recruit from lists, and patients were offered one‐off participation in a focus group. They found divergence between patient and research council priorities for research and stated that the process added insight, but it is not clear whether the information was actually used.

Lee^48^ revised medication information leaflets by working with pharmacies to recruit patients to individual interviews where they appraised readability and content of information. The information went through several reviews using a consumer involvement framework, and the user recommendations were used by researchers to develop Consumer Medication Information.

The different approaches to targeted involvement suggested that frequent involvement establishes/strengthens relationships and iterative discussions can produce consensus on research priorities as well as useful patient information. In contexts where involvement is used to extract information via a one‐off encounter (Brown 2006), the utility of the involvement remains unclear.

A second set of projects characterized as embedded involvement.^85^^,^ 86 used semi‐structured focus groups and interviews at several points to inform design of an intervention and materials. Researchers retained decision‐making authority and control. Repeated contact with the same set of service users was not reported. They concluded that participation by patients and general practices appeared to be inadequate and suggested that there was a ‘need to rethink context and the hierarchical relationships between children, young people, parents and professionals with regard to “partnership and participation” in diabetes decision‐making, self‐care and self‐management’^86^:xxxv.

This led us to explore contexts where the pre‐existing relationships, that were initially hierarchical, subsequently shifted to shared control. Lindenmeyer^49^ retrospectively assessed user contributions in an established Research User Group. Co‐learning, training and support over time served to clarify the purpose of involvement for both users and researchers. Researchers acknowledged that it was challenging to relinquish control but agreed that users changed the direction of the research studies, adding credibility to proposals, making them more likely to be funded, and producing interventions that were more relevant to people with diabetes. This indicated that in contexts where researchers are able to relinquish control, reciprocal relationships can emerge, triggering mechanisms where users feel confident to contribute knowledge, thereby increasing the relevance of the research.

The relationship between reciprocal relationships and relevance also appeared in projects that focused on developing educational materials. For example, Evans^45^ actively engaged primary care staff in an action research project where they iteratively developed more appropriate educational materials for supporting people with pre‐diabetes. Their rationale for action research was that it enables ‘the developmental process to be grounded in the views of both service users and frontline staff’^45^:771. The materials were co‐developed, tried with patients and revised based on feedback from both patients and staff. This ongoing process of exchange produced relevant, acceptable and useful educational resources for people with diabetes and professionals.

The regular and reciprocal nature of the relationship is an explicit requirement in research involving Aboriginal people, as was noted in Shoen^51^ and Watson^53^. Reciprocity is defined in Aboriginal research as the requirement that researchers must demonstrate a return for participation that is of benefit to the community. In Schoen's project, this took the form of reciprocal interventions such as foot clinics, diabetes training and education while Watson's team produced educational materials that were owned by the Aboriginal participants. Reciprocal dialogue, where the knowledge of local people was given equal value, enabled the identification of issues that were important with people who had diabetes. Outcomes for collaborative production of educational materials included a more coordinated team approach with clearer strategies for supporting patients with pre‐diabetes management^45^; co‐developed materials, which increased understanding of diabetes risk and were incorporated into support services, training and manuals that were useful in informing people with diabetes, other community members and professionals^50^, ^51^, ^52^, ^53^.

Although initial involvement may inform project design, researcher control over subsequent stages of projects may undermine initial co‐development. Two projects that consulted patients and the wider community to inform research design, Peer Support in Diabetes^118^^‐^ 119 and RAPSID^120^, ^121^, ^122^ maintained control over recruitment and content of the intervention. Patient recruitment was low, and both projects found that peer supporters who were recruited dropped out. The RAPSID project noted the need to recruit peer supporters directly rather than through clinicians and that recruitment to trials requires very careful preparation, management and an understanding of the population involved. We compared these projects with another set that maintained user relationships through the stages of recruitment and delivery of the intervention.

### 3.1 Collaboration and coproduction across all stages of the research

Projects reporting involvement across all stages collaborated to explore local environments, design and conduct interventions. These projects noted that developing partnerships took time, in terms of making use of the very different areas of expertise held by patients, the public and researchers^44^^,^ 47. Partnerships incorporated local people who were committed to reducing health disparity^101^, ^102^, ^103^, ^104^, ^105^ , with reputations as ‘doers and consensus makers’. Task groups included people from community agencies and universities who met regularly and reported back to Community Advisory Boards (CAB)^114^^‐^ 115 ^,^ 123 ^‐^ 124. The Advisory Boards had project oversight as well as facilitating acceptance and participation in the research via their local social networks, sectors and organizations. In some places, the Advisory Board was established first in order to set priorities for the research^101^, ^102^, ^103^, ^104^, ^105^  , while in other settings, the project was initiated by academics^133^^‐^ 134. In the five projects that were conducted in First Nation settings (British Columbia^65^; FEDS^87^, ^88^, ^89^, ^90^; HCSF^91^, ^92^, ^93^, ^94^, ^95^, ^96^, ^97^, ^98^, ^99^, ^100^; Kahnawake^106^, ^107^, ^108^, ^109^, ^110^, ^111^, ^112^, ^113^; Sandy Lake^125^, ^126^, ^127^, ^128^, ^129^, ^130^) the Tribal Councils had legal authority over decisions to conduct research on the reservations, which placed academics in a position of making a formal application to work with communities.

Partnerships were tasked with developing collaborative relationships via regular face‐to‐face open meetings and site visits in order to enable members to express concerns and generate culturally appropriate solutions^114^^‐^ 115 ^,^ 123 ^‐^ 124. Communication issues were addressed via different group techniques that were used to develop solidarity, a shared purpose and a shared knowledge base^101^, ^102^, ^103^, ^104^, ^105^ .

Partnerships started with an exploration of how local environment and contexts influenced perceptions of diabetes and challenged self‐management. Data were collected to document prevalence and raise awareness of risk 91 ^‐^ 92 ^,^ 106 ^,^ 116; combined with qualitative work to explore perceptions of risk and diabetes^57^,^76^^,^ 87 ^,^ 94 ^,^ 101 ^,^ 123 ^,^ 125, 126, 127 ^,^ 131 ^,^ 133. This exploration led to an increased awareness that diabetes was a problem that needed to be addressed.

Partnership governance was instrumental in making shared decisions, and negotiating differential skills and expertise. Co‐design and training for local researchers increased skills^55^^,^ 57.

Partnerships felt that the engagement and empowerment of people in defining and finding solutions to diabetes for their community lead to a sense of responsibility and control over programmes, which should theoretically promote change^58^^,^ 108 ^,^ 133 ^‐^ 134.

Co‐learning from exploration of local context was used to develop theory‐based protocols that were culturally acceptable, producing accessible and efficacious interventions^89^^‐^ 90 ^,^ 123 ^‐^ 124. Using local facilitators for interviews and focus groups meant that people could freely express opinions, which was key to considering acceptable interventions^77^^,^ 82 ^,^ 131.

The feasibility of making lifestyle changes was considered in relation to cultural norms and local resources. For example, the availability and cost of food was mapped and used to leverage local resources to provide healthier and more affordable choices^102^^,^ 129. In some projects, one approach to intervention development was tried and evaluated before revising^114^^‐^ 115 or moving on to another strategy^95^, ^96^, ^97^, ^98^, ^99^, ^100^ while others adopted a multifaceted approach which included a number of local activities which ran in tandem^72^, ^73^, ^74^^,^ 87, 88, 89, 90 ^,^ 130. Responsiveness to local conditions was noted to be key in designing interventions^107^.

Materials and approaches to supporting people with diabetes and the wider community drew upon local values, lifestyles and traditional social structures^60^^,^ 90 ^,^ 123 ^,^ 130. Local workers and diabetes patients tailored educational teaching style and messages, incorporating components that culturally resonate^50^, ^51^, ^52^, ^53^^,^ 56 ^,^ 62 ^,^ 70 ^,^ 73 ^,^ 79 ^,^ 81 ^,^ 83 ^,^ 84 ^,^ 93 ^,^ 95 ^,^ 103 ^,^ 117 ^,^ 124 ^,^ 131 ^,^ 133. Co‐created information ensured cultural appropriateness, respect for local practices and feasibility and acceptability of advice. Community presence contributed to the design of effective and efficient recruitment protocols and ensured the acceptability of the methods to potential participants^55^^,^ 104. This was associated with high levels of satisfaction with programmes^124^.

Projects that aimed to co‐design controlled trials encountered a number of challenges. Funders found it difficult to accept that there were ethical issues to using a control group design^117^. Time periods of 3‐14 years were needed to agree the methods and intervention (Alabama Black Belt; Detroit REACH; HCSF; HEED; LLDPP; Yakima Valley). Development was affected by historical relationships between researchers and communities. In communities that distrusted research, it took longer to establish relationships (Detroit REACH; HCSF). Where longer periods of time were needed, projects were placed in a position of having to sustain activity while applying for a series of funding proposals.

Local coordinators and agencies who had long‐standing relationships with people and were respected in the community were instrumental in managing trials^56^^,^ 104 ^‐^ 105 ^,^ 114 ^‐^ 115 ^,^ 124 ^,^ 131 ^‐^ 132. Hiring and retaining qualified local people (site coordinators; community health workers; peer support workers) was a challenge, but recruitment/retention teams were an important component of study design.

Trials used inclusive approaches to establishing control groups. There was randomization at household level, with additional family members assigned to the same intervention^55^^,^ 115, and active control groups where all families received a version of the intervention^99^^‐^ 100. Stepped recruitment successfully retained control group participation because people believed that they would eventually receive the intervention^132^. Ensuring sustained participation, however, incurred more costs (time, money) as a result.

High rates of recruitment and retention were attributed to locally led recruitment teams who were sensitive to neighbourhood constraints and possibilities, and who were involved at proposal, recruitment and data collection stages^55^^‐^ 56 ^,^ 100 ^,^ 115,124 ^,^ 132. The expertise and connections of respected community agencies promoted trust and acceptability, ‘which likely decreased participant withdrawal from the study’^124^:363.

Local contexts challenged sustainability of interventions. A number of the projects noted the challenges of following dietary guidelines when people live in areas where it is difficult and expensive to get healthy food options (British Columbia, Chicago REACH, Kahnawake). Other projects noted that exercise was difficult due to the lack of exercise facilities, paved paths, safety risks and inclement weather (HCSF; Sandy Lake; Starr County). Increase in disposable income and childcare mitigated against the initial positive indications of healthier eating^109^. Partnerships were challenged when resources (both human and financial) are thinly stretched across a range of competing needs in high‐deprivation communities^130^.

### What contributes to the success or failure of involvement?

Theories about involvement^5^, ^6^, ^31^, ^32^, ^33^ and project data were synthesized to construct a series of propositions for how involvement works, in what contexts, for whom, at different stages of design and implementation of the intervention (Table 2).

**Table 3:** Propositions about involvement in diabetes research

Stage of research project	Relationship between context, mechanisms and outcomes
Priority setting	When setting priorities, the involvement of people with experience of diabetes *may* increase relevance but *only if* there is a context where: there is productive and sufficiently long interaction between researchers and patients/communities there is facilitation and/or training providing opportunities for people to share knowledge and experiences the people involved are similar or the same as those that will be designing the intervention
	When these conditions exist, mechanisms are triggered where: researchers aware of their own stance and are willing to relinquish control community members feel safe to share concerns and disagree people commit to working out differences
	The outcomes of the priority setting process are as follows: problem framing which allows patient and community concerns to be foregrounded agreed topics that are taken to funding bodies raised awareness of health issues and possible solutions that are relevant to patients/communities continued interest in participating in design and mobilizing to take action
Design of the intervention	During the design stage, the same context is important, with the added provisos that: relevant stakeholders and agencies need to be included stakeholders need experience in facilitating partnership working safe and comfortable spaces need to be identified to encourage participation of new stakeholders
	If these conditions are in place, then members of the project group will feel: empowered to judge the feasibility and appropriateness of the emerging design clear on who has the expertise to undertake different study tasks
	If successful, the end products are more culturally acceptable interventions, more appropriate approaches to recruitment, and more user‐friendly information and tools. Excluding people from the design process may lead to project information that is difficult to understand and less culturally acceptable
Implementation stage	Implementing an intervention needs some key resources, which include the following: training for local people and community workers that is appropriate for their needs and levels of knowledge supportive supervision for people who are explaining the project to potential participants, providing the intervention, collecting and analysing data knowledge of the ways in which different stakeholders can contribute to the initiative When these resources are in place, the people delivering the interventions feel empowered to exercise judgement when recruiting to the study, confident to tailor the intervention based on patient/community needs and concerns, comfortable to raise issues about acceptability and suggesting how components can be modified

### 3.2 Exploring relationships between the process of involvement and health outcomes

Our final review question asked: Does successful involvement in adapting diabetes interventions benefit people with diabetes, communities and practitioners lead to achievement of health outcomes?

We assessed the impact of involvement on the research process as well as on health outcomes. A participatory research impact framework^34^ was used to chart reported benefits (Table 4).

**Table 4:** Reported benefits of involvement

Benefit	Reported
On research agenda	
Initiating the research topic	R
Identifying different research questions	R
Influencing funding decisions	NR
On research design	
Amending the focus of this research	R
Shaping the question (s) for this research	R
Designing data collection/generation approach	R
Designing approach to data analysis	R
Increased trustworthiness	R
Quality of the data	R
On research process	
Supporting recruitment	R
Collecting/Generating data	R
Analysing data	R
Writing up	NR
Dissemination	NR
On participatory researchers	
Knowledge of research	R
Confidence to contribute	R
Skills	R
Empowerment	R
On academic/community based researchers	
Affected perceptions	R
Affected engagement with communities of practice	R
Affected understandings of topic area	R

Note

Adapted from Cook et al.^34^

Projects agreed that successful inclusion of people influenced decisions about research priorities and topics. Projects that consistently used involvement described it as having a cumulative effect. Community engagement in framing the problem increases interest in co‐designing an intervention. The focus of the research is amended, producing research questions and methods that are shaped to local contexts and circumstances. Inclusion in collecting data and analysing it increases the trustworthiness of the findings and the quality of the data. Involvement of local people in recruitment enables projects to achieve high rates and involvement of local people in delivering interventions contributes to sustained retention. Benefits to community researchers include increased knowledge and confidence to contribute to different stages of the project. Academic researchers reported that their perceptions of the utility of involvement changed and they developed skills in engaging with communities of practice and an increased understanding of the topic. Although involvement was seen to benefit the process at a number of steps, participation in data analysis, writing up and dissemination were rarely reported.

Projects using a collaborative approach related involvement to achieving health outcomes. Cohesive collaborative partnerships were seen to impact local community health (HEED) and culturally relevant programmes which were sensitive to literacy were associated with efficacy in diabetes management (San Francisco) evidenced by improved HbA1c, weight loss and improved insulin resistance (LLDPP). Conversely, projects that were unable to embed involvement or mobilize partnerships were unable to show clinical effect (British Columbia; EPIC; PSPD; RAPSID;).

## 4 DISCUSSION AND CONCLUSIONS

This review found that projects which promote frequent and regular contact across researchers, patients with diabetes and wider communities are able to develop reciprocal relationships where the lived experiences of people are instrumental in developing and conducting relevant and accessible interventions promoting diabetes self‐management. In contexts where researchers are able to share control and ownership, community advisory groups and community researchers can achieve and sustain high rates of participation, which can potentially support achievement of health outcomes.

Although we were able to follow our original protocol,^35^ our theory depended on the extent of explanation and attribution in papers reporting the process of involvement. We compensated for thin reporting by using cluster searching to identify additional reports for each project. Our finding that good rates of recruitment and co‐development of recruitment influence high uptake and retention rates is consistent with a review by Horigan et al^36^ that found non‐attendance occurs when these factors are not addressed. Our finding that delivery by local workers influences retention is consistent with trials noting that community health worker intervention groups have higher completion rates than usual care groups.^37^ The importance of reciprocity is supported by a realist review^38^ and a validated model for community‐based participatory research that demonstrate the importance of relationships when working in academic/community partnerships to achieve individual and system outcomes.^39^ To the best of our knowledge, there are no other reviews attempting to link involvement in diabetes interventions to health outcomes, but there is strong evidence across a range of health conditions that community engagement is effective.^40^

However, involvement requires investment in new ways of working, which research funding rarely covers. The development of partnership trust takes time, as was acknowledged by a number of the teams included in the current review. Therefore, we need to ask how limited resources can best be used to incorporate patient and community experiences into diabetes research. We need to define what ‘good enough’ involvement actually means; in some contexts, targeted engagement may be sufficient, while in others, ongoing involvement and active collaboration may be needed.

It has been argued that research funders currently prioritize research designs that favour controlled studies at the expense of ignoring complexity in health interventions.^41^ Further, in the case of diabetes, research projects are generally expected to generate evidence of clinical impact over a relatively short time period of 6‐18 months. The successful projects in our review placed health outcomes within a wider and longer term perspective and defined impact as achieving understanding of community issues and context, developing trusting relationships across sectors and developing productive partnerships. These dimensions of impact are prerequisites for designing research that is feasible and locally relevant as well as robust.^42^

Research teams need to incorporate the key aspects of working in partnerships and community development that were identified in our review. In addition, more support needs to be dedicated to funding different types of research that foreground interactions, interconnectedness and understanding of how integral community systems are to reduction of diabetes risk and self‐management of diabetes.

## CONFLICTS OF INTEREST

The authors confirm that they have no conflicts of interest.

## Data Availability

Data sharing is not applicable to this article as no new data were created or analysed in this review.
