# Supplementary material for: How patient and community involvement in diabetes research influences health outcomes: A realist review
Source: Health Expect. 2019 Jul 8;22(5):907–20. doi: 10.1111/hex.12935 (PMC6803418; doi:10.1111/hex.12935)
Supplement: Supplementary file 1 [file HEX-22-907-s001.docx]

**Supporting Information**

**INCLUDED PROJECTS Targeted and Embedded Involvement**

Brown K, Dyas J, Chahal P, Khalil Y, Riaz P, Cummings-Jones J. Discovering the research priorities of people with diabetes in a multicultural community: a focus group study. Br J Gen Pract. 2006;56(524):206-13.

Carlson BA, Neal D, Magwood G, Jenkins C, King MG, Hossler CL. A community-based participatory health information needs assessment to help eliminate diabetes information disparities. Health promot pract. 2006;7(3 suppl):213S-22S.

Evans PH, Greaves C, Winder R, Fearn‐Smith J, Campbell JL. Development of an educational ‘toolkit’ for health professionals and their patients with prediabetes: The WAKEUP study (Ways of Addressing Knowledge Education and Understanding in Pre‐diabetes). Diabet Med. 2007;24(7):770-7.

Gadsby R, Snow R, Daly AC, Crowe S, Matyka K, Hall B, Petrie J. Setting research priorities for Type 1 diabetes. Diabet Med. 2012 Oct 1;29(10):1321-6.

Giachello AL, Arrom JO, Davis M, Sayad JV, Ramirez D, Nandi C, Ramos C. Reducing diabetes health disparities through community-based participatory action research: the Chicago Southeast Diabetes Community Action Coalition. Public health rep. 2003;118(4):309-23.

Lee DY, Armour C, Krass I. The development and evaluation of written medicines information for Type 2 diabetes. Health Educ Res. 2007;22(6):918-30.

Lindenmeyer A, Hearnshaw H, Sturt J, Ormerod R, Aitchison G. Assessment of the benefits of user involvement in health research from the Warwick Diabetes Care Research User Group: a qualitative case study. Health Expect. 2007 10(3):268-77.

Mudd-Martin G, Martinez MC, Rayens MK, Gokun Y, Meininger JC. Sociocultural Tailoring of a Healthy Lifestyle Intervention to Reduce Cardiovascular Disease and Type 2 Diabetes Risk Among Latinos. Prev Chronic Dis. 2013;10:130-137.

Schoen D, Balchin D, Thompson S. Health promotion resources for Aboriginal people: lessons learned from consultation and evaluation of diabetes foot care resources. Health Promot J Austr. 2010;21(1):64-9.

Thompson SJ, Gifford SM, Thorpe L. The social and cultural context of risk and prevention: Food and physical activity in an urban Aboriginal community. Health Educ Behav. 2000 Dec;27(6):725-43.

Watson J, Obersteller EA, Rennie L, Whitbread C. Diabetic foot care: developing culturally appropriate educational tools for Aboriginal and Torres Strait Islander peoples in the Northern Territory, Australia. Aust J Rural Health. 2001;9(3):121-6.

**INCLUDED PROJECTS Collaborative and User-led Research**

**Alabama Black Belt**

Cherrington A, Martin MY, Hayes M, Halanych JH, Andreae SJ, Safford M, Wright MA, Appel SJ. Intervention mapping as a guide for the development of a diabetes peer support intervention in rural Alabama. Prev Chronic Dis. 2012;9:110053. http://dx.doi.org/10.5888/pcd9.110053 .

Andreae SJ, Halanych JH, Cherrington A, Safford MM. Recruitment of a rural, southern, predominantly African-American population into a diabetes self-management trial. Contemp clin trials. 2012;33(3):499-506.

Safford MM, Andreae S, Cherrington AL, Martin MY, Halanych J, Lewis M, Patel A, Johnson E, Clark D, Gamboa C, Richman JS. Peer coaches to improve diabetes outcomes in rural Alabama: a cluster randomized trial. The Ann Fam Med. 2015;13(Suppl 1):S18-26.

**American Samoa**

Braun KL, Kuhaulua RL, Ichiho HM, Aitaoto NT. Listening to the community: a first step in adapting Diabetes Today to the Pacific. Pac Health Dialog. 2002;9(2):321-8.

Braun KL, Ichiho HM, Kuhaulua RL, Aitaoto NT, Tsark JU, Spegal R, Lamb BM. Empowerment through community building: Diabetes Today in the Pacific. J **Public Health** Manag Pract. 2003;9:S19-25.

Aitaoto NT, Braun KL, Ichiho HM, Kuhau RL. Diabetes Today in the Pacific: reports from the field. Pac Health Dialog. 2005;12(1):124-31.

Rosen RK, DePue J, McGarvey ST. Overweight and diabetes in American Samoa: the cultural translation of research into health care practice. Med Health R I. 2008;91(12):372-377.

Aitaoto N, Tsark J, Braun KL. Sustainability of the Pacific Diabetes Today coalitions. Prev Chronic Dis. 2009;6(4):A130. Available from http://www.cdc.gov/pcd/issues/2009/oct/08_0181.htm. [Cited 30 July 2018]

DePue JD, Dunsiger S, Seiden AD, Blume J, Rosen RK, Goldstein MG, Nu'usolia O, Tuitele J, McGarvey ST. Nurse–community health worker team improves diabetes care in American Samoa. Diabetes Care. 2013;36(7):1947-53.

Hamid S, Dunsiger S, Seiden A, Nu'usolia O, Tuitele J, DePue JD, McGarvey ST. Impact of a diabetes control and management intervention on health care utilization in American Samoa. Chronic Illn. 2014;10(2):122-34.

Rao M, DePue JD, Dunsiger S, Elsayed M, Nu'usolia O, McGarvey ST. Long-Term Impact of a Community Health Worker Intervention on Diabetes Control in American Samoa. Prev Chronic Dis. 2015;12:150160. Available from: http:// dx.doi.org/10.5888/pcd12.150160.

**British Columbia**

Daniel M, Green LW, Marion SA, Gamble D, Herbert CP, Hertzman C, Sheps SB. Effectiveness of community-directed diabetes prevention and control in a rural Aboriginal population in British Columbia, Canada. Soc Sci Med. 1999 Mar 31;48(6):815-32.

**Chicago REACH**

Peek ME, Quinn MT, Gorawara-Bhat R, Odoms-Young A, Wilson SC, Chin MH. How is shared decision-making defined among African-Americans with diabetes? Patient Educ Couns. 2008;72(3):450-8.

Peek ME, Wilson SC, Gorawara-Bhat R, Odoms-Young A, Quinn MT, Chin MH. Barriers and facilitators to shared decision-making among African-Americans with diabetes. J General Intern Med. 2009;24(10):1135-1139.

Peek ME, Odoms-Young A, Quinn MT, Gorawara-Bhat R, Wilson SC, Chin MH. Race and shared decision-making: perspectives of African-Americans with diabetes. Soc Sci Med. 2010;71(1):1-9.

Peek ME, Wilkes AE, Roberson TS, Goddu AP, Nocon RS, Tang H, Quinn MT, Bordenave KK, Huang ES, Chin MH. Early lessons from an initiative on Chicago’s South Side to reduce disparities in diabetes care and outcomes. Health Aff. 2012;31(1):177-86.

Peek ME, Harmon SA, Scott SJ, Eder M, Roberson TS, Tang H, Chin MH. Culturally tailoring patient education and communication skills training to empower African-Americans with diabetes. Transl Behavioral Med. 2012;2(3):296-308.

Nundy S, Dick JJ, Solomon MC, Peek ME. Developing a behavioral model for mobile phone-based diabetes interventions. Patient Educ Couns. 2013;90(1):125-32.

Chin MH, Goddu AP, Ferguson MJ, Peek ME. Expanding and sustaining integrated health care – Community efforts to reduce diabetes disparities. Health Promot Pract. 2014;15(2_suppl):29S-39S.

Peek ME, Ferguson MJ, Roberson TP, Chin MH. Putting theory into practice: a case study of diabetes-related behavioral change interventions on Chicago’s south side. Health Promot Pract. 2014;15(2_suppl):40S-50S.

Goddu AP, Roberson TS, Raffel KE, Chin MH, Peek ME. Food Rx: a community–university partnership to prescribe healthy eating on the South Side of Chicago. J Prevention Interv Community. 2015;43(2):148-62.

Goddu AP, Raffel KE, Peek ME. A story of change: The influence of narrative on African-Americans with diabetes. Patient Educ Couns. 2015;98(8):1017-24.

**Detroit REACH**

Kieffer EC, Willis SK, Odoms-Young AM, Guzman JR, Allen AJ, Feathers JT, Loveluck J. Reducing disparities in diabetes among African-American and Latino residents of Detroit: the essential role of community planning focus groups. Ethnicity and Disease. 2004;14(3; SUPP/1):S1-27.

Feathers JT, Kieffer EC, Palmisano G, Anderson M, Sinco B, Janz N, Heisler M, Spencer M, Guzman R, Thompson J, Wisdom K. Racial and Ethnic Approaches to Community Health (REACH) Detroit partnership: improving diabetes-related outcomes among African American and Latino adults. Am J Public Health. 2005;95(9):1552-60.

Spencer MS, Kieffer EC, Sinco BR, Palmisano G, Guzman JR, James SA, Graddy-Dansby G, Feathers JT, Heisler M. Diabetes-specific emotional distress among African Americans and Hispanics with type 2 diabetes. J Health Care Poor Underserved, 2006;17(2):88-105.

Two Feathers J, Kieffer EC, Palmisano G, Anderson M, Janz N, Spencer MS, Guzman R, James SA. The development, implementation, and process evaluation of the REACH Detroit Partnership's Diabetes Lifestyle Intervention. Diabetes Educ. 2007;33(3):509-20.

Rosland AM, Kieffer E, Israel B, Cofield M, Palmisano G, Sinco B, Spencer M, Heisler M. When is social support important? The association of family support and professional support with specific diabetes self-management behaviors. J General Intern Med. 2008;23(12):1992-1999.

Heisler M, Spencer M, Forman J, Robinson C, Shultz C, Palmisano G, Graddy-Dansby G, Kieffer E. Participants' assessments of the effects of a community health worker intervention on their diabetes self-management and interactions with healthcare providers. Am J Prev Med. 2009;37(6):S270-9.

Spencer MS, Rosland AM, Kieffer EC, Sinco BR, Valerio M, Palmisano G, Anderson M, Guzman JR, Heisler M. Effectiveness of a community health worker intervention among African American and Latino adults with type 2 diabetes: a randomized controlled trial. Am J Public Health. 2011;101(12): 2253-60.

Tang TS, Funnell M, Sinco B, Piatt G, Palmisano G, Spencer MS, Kieffer EC, Heisler M. Comparative effectiveness of peer leaders and community health workers in diabetes self-management support: results of a randomized controlled trial. Diabetes Care. 2014;37(6):1525-34.

Spencer MS, Kieffer EC, Sinco B, Piatt G, Palmisano G, Hawkins J, Lebron A, Espitia N, Tang T, Funnell M, Heisler M. Outcomes at 18 Months From a Community Health Worker and Peer Leader Diabetes Self-Management Program for Latino Adults. Diabetes Care. 2018;41:1414-1422.

**EPIC Project**

Noyes JP, Williams A, Allen D, Brocklehurst P, Carter C, Gregory JW, Jackson C, Lewis M, Lowes L, Russell IT, Rycroft-Malone J. Evidence into practice: evaluating a child-centred intervention for diabetes medicine management The EPIC Project. BMC Pediatr. 2010;10(1):70.

Noyes JP, Lowes L, Whitaker R, Allen D, Carter C, Edwards RT, Rycroft-Malone J, Sharp J, Edwards D, Spencer LH, Sylvestre Y. Developing and evaluating a child-centred intervention for diabetes medicine management using mixed methods and a multicentre randomised controlled trial. Health Serv Deliv Res. 2014;2:(8).

**Family Education Diabetes Series (FEDS)**

Doherty WJ, Mendenhall TJ. Citizen health care: A model for engaging patients, families, and communities as coproducers of health. Fam Sys Health. 2006;24(3):251-263.

Berge JM, Mendenhall TJ, Doherty WJ. Using Community‐Based Participatory Research (CBPR) to Target Health Disparities in Families. Fam Relat. 2009;58(4):475-88.

Mendenhall TJ, Berge JM, Harper P, GreenCrow B, LittleWalker N, WhiteEagle S, BrownOwl S. The Family Education Diabetes Series (FEDS): community‐based participatory research with a midwestern American Indian community. Nurs Inq. 2010;17(4):359-72.

Mendenhall TJ, Seal KL, GreenCrow BA, LittleWalker KN, BrownOwl SA. The family education diabetes series: Improving health in an urban-dwelling American Indian community. Qual Health Res. 2012;22(11):1524-34.

**Healthy Children Safe Families (HCSF)**

Adams A, Miller-Korth N, Brown D. Learning to work together: developing academic and community research partnerships. Wis Med J. 2004;103(2):15–9.

Adams AK, Quinn RA, Prince RJ. Low recognition of childhood overweight and disease risk among Native-American caregivers. Obes Res. 2005;13:146–52.

LaRowe TL, Wubben DP, Cronin KA, Adams AK, Vannatter SM. Peer Reviewed: Development of a Culturally Appropriate, Home-Based Nutrition and Physical Activity Curriculum for Wisconsin American Indian Families. Prev Chronic Dis. 2007 Oct;4(4).

Adams AK, Harvey H, Brown D. Constructs of health and environment inform child obesity prevention in American Indian communities. Obesity. 2008 Feb 1;16(2):311-7.

Adams A. Understanding community and family barriers and supports to physical activity in American Indian children. J **Public Health** Manag Pract. 2010;16(5):401-403.

Adams A, Prince R. Correlates of Physical Activity in Young American Indian Children: Lessons Learned from the Wisconsin Nutrition and Growth Study (WINGS). J **Public Health** Manag Pract. 2010;16(5):394-400.

Adams AK, LaRowe TL, Cronin KA, Prince RJ, Wubben DP, Parker T, Jobe JB. The Healthy Children, Strong Families intervention: design and community participation. J Prim Prev. 2012;33(4):175-85.

Adams AK, Scott JR, Prince R, Williamson A. Peer Reviewed: Using Community Advisory Boards to Reduce Environmental Barriers to Health in American Indian Communities, Wisconsin, 2007–2012. Prev Chronic Dis. 2014;11.

Tomayko EJ, Prince RJ, Cronin KA, Adams AK. The Healthy Children, Strong Families intervention promotes improvements in nutrition, activity and body weight in American Indian families with young children. Public Health Nutr. 2016:1-0.

Tomayko EJ, Prince RJ, Cronin KA, Parker T, Kim K, Grant VM, Sheche JN, Adams AK. Healthy Children, Strong Families 2: A randomized controlled trial of a healthy lifestyle intervention for American Indian families designed using community-based approaches. Clin Trials. 2017:19(15):2850-2859.

**HEED East Harlem**

Horowitz CR, Williams L, Bickell NA. A community-centred approach to diabetes in East Harlem. J General Intern Med. 2003;(18):542-8.

Horowitz CR, Colson KA, Hebert PL, Lancaster K. Barriers to buying healthy foods for people with diabetes: evidence of environmental disparities. Am J Public Health. 2004;94(9):1549-54.

Goldfinger JZ, Arniella G, Wylie-Rosett J, Horowitz CR. Project HEAL: peer education leads to weight loss in Harlem. J Health Care Poor Underserved. 2008;19(1):180-192.

Horowitz CR, Brenner BL, Lachapelle S, Amara DA, Arniella G. Effective recruitment of minority populations through community-led strategies. Am J Prev Med. 2009;37(6):S195-200.

Horowitz CR, Eckhardt S, Talavera S, Goytia C, Lorig K. Effectively translating diabetes prevention: a successful model in a historically underserved community. Transl Behav Med. 2011;1(3):443.

**Kahnawake Schools Diabetes Prevention Project**

Macaulay AC, Paradis G, Potvin L, Cross EJ, Saad-Haddad C, McComber A, Desrosiers S, Kirby R, Montour LT, Lamping DL, Leduc N. The Kahnawake Schools Diabetes Prevention Project: intervention, evaluation, and baseline results of a diabetes primary prevention program with a native community in Canada. Prev Med 1997;26(6):779-90.

Potvin L, Cargo M, McComber AM, Delormier T, Macaulay AC. Implementing participatory intervention and research in communities: lessons from the Kahnawake Schools Diabetes Prevention Project in Canada. Soc Sci Med. 2003;56(6):1295-305.

Bisset S, Cargo M, Delormier T, Macaulay AC, Potvin L. Legitimizing diabetes as a community health issue: a case analysis of an Aboriginal community in Canada. Health Promot Int. 2004;19(3):317-26.

Paradis G, Lévesque L, Macaulay AC, Cargo M, McComber A, Kirby R, Receveur O, Kishchuk N, Potvin L. Impact of a diabetes prevention program on body size, physical activity, and diet among Kanien'keha: ka (Mohawk) children 6 to 11 years old: 8-year results from the Kahnawake Schools Diabetes Prevention Project. Pediatr. 2005;115(2):333-9.

Macaulay AC, Ing A, Salsberg J, McGregor A, Saad-Haddad C, Rice J, Montour L, Gray-Donald K. Community-based participatory research: lessons from sharing results with the community: Kahnawake schools diabetes prevention project. Prog Community Health partnersh. 2007;1(2):143-52.

Cargo M, Delormier T, Lévesque L, Horn-Miller K, McComber A, Macaulay AC. Can the democratic ideal of participatory research be achieved? An inside look at an academic–indigenous community partnership. Health Ed Res. 2008;23(5):904-14.

Cargo MD, Delormier T, Lévesque L, McComber AM, Macaulay AC. Community capacity as an “inside job”: evolution of perceived ownership within a university-aboriginal community partnership. Am J Health Promot. 2011;26(2):96-100.

Salsberg J, Macridis S, Garcia Bengoechea E, Macaulay AC, Moore S, KSDPP School Travel Planning Committee. The shifting dynamics of social roles and project ownership over the lifecycle of a community-based participatory research project. Fam Pract. 2017;34(3):305-12.

**Lawrence Latinos Diabetes Prevention Project (LLDPP)**

Merriam PA, Tellez TL, Rosal MC, Olendzki BC, Ma Y, Pagoto SL, Ockene IS. Methodology of a diabetes prevention translational research project utilizing a community-academic partnership for implementation in an underserved Latino community. BMC Med Res Methodol. 2009;9(1):20.

Ockene IS, Tellez TL, Rosal MC, Reed GW, Mordes J, Merriam PA, Olendzki BC, Handelman G, Nicolosi R, Ma Y. Outcomes of a Latino community-based intervention for the prevention of diabetes: the Lawrence Latino Diabetes Prevention Project. Am J Public Health. 2012;102(2):336-42.

**New Zealand Ngati**

Coppell KJ, Tipene-Leach DC, Pahau HL, Williams SM, Abel S, Iles M, Hindmarsh JH, Mann JI. Two-year results from a community-wide diabetes prevention intervention in a high risk indigenous community: the Ngati and Healthy project. Diabetes Res Clin Pract. 2009;85(2):220-7.

Tipene-Leach DC, Coppell KJ, Abel S, Pāhau HL, Ehau T, Mann JI. Ngāti and healthy: translating diabetes prevention evidence into community action. Ethn Health. 2013;18(4):402-14.

**Peer Support for People with Type 2 Diabetes (PSPD)**

Paul G, Smith SM, Whitford D, O'Kelly F, O'Dowd T. Development of a complex intervention to test the effectiveness of peer support in type 2 diabetes. BMC Health Serv Res 2007;7(1):136.

Smith SM, Paul G, Kelly A, Whitford DL, O’Shea E, O’Dowd T. Peer support for patients with type 2 diabetes: cluster randomised controlled trial. BMJ. 2011 Feb 15;32:d715.

**RAPSID Peer Support in Diabetes**

Simmons D, Bunn C, Cohn S, Graffy J. What is the idea behind peer to peer support in diabetes. Diabetes Manag. 2013;3(1):61-70.

Simmons D, Cohn S, Bunn C, Birch K, Donald S, Paddison C, Ward C, Robins P, Prevost AT, Graffy J. Testing a peer support intervention for people with type 2 diabetes: a pilot for a randomised controlled trial. BMC Fam Pract 2013;14(1):5.

Simmons D, Prevost AT, Bunn C, Holman D, Parker RA, Cohn S, Donald S, Paddison CA, Ward C, Robins P, Graffy J. Impact of community based peer support in type 2 diabetes: a cluster randomised controlled trial of individual and/or group approaches. PLoS One. 2015;10(3):e0120277.

**San Francisco**

Chesla CA, Chun KM, Kwan CM. Cultural and family challenges to managing type 2 diabetes in immigrant Chinese Americans. Diabetes Care. 2009;32(10):1812-6.

Chesla CA, Chun KM, Kwan CM, Mullan JT, Kwong Y, Hsu L, Huang P, Strycker LA, Shum T, To D, Kao R. Testing the efficacy of culturally adapted coping skills training for Chinese American immigrants with type 2 diabetes using community‐based participatory research. Res Nurs Health. 2013;36(4):359-72.

**Sandy Lake**

Hanley AJ, Harris SB, Barnie A, Gittelsohn J, Wolever TM, Logan A, Zinman B. The Sandy Lake Health and Diabetes Project: design, methods and lessons learned. Chronic Dis Can. 1995;16(4):149-56.

Gittelsohn J, Harris SB, Whitehead S, Wolever TM, Hanley AJ, Barnie A, Kakegamic L, Logan A, Zinman B. Developing diabetes interventions in an Ojibwa-Cree community in northern Ontario: Linking qualitative and quantitative data. Chronic Dis Can. 1995;16(4):157-64.

Gittelsohn J, Harris SB, Burris KL, Kakegamic L, Landman LT, Sharma A, Wolever TM, Logan A, Barnie A, Zinman B. Use of ethnographic methods for applied research on diabetes among the Ojibway-Cree in northern Ontario. Health Educ Behav. 1996;23(3):365-82.

Gittelsohn J, Harris SB, Thorne-Lyman AL, Hanley AJ. Body image concepts differ by age and sex in an Ojibway-Cree community in Canada. J Nutr. 1996;126(12):2990.

Saksvig BI, Gittelsohn J, Harris SB, Hanley AJ, Valente TW, Zinman B. A pilot school-based healthy eating and physical activity intervention improves diet, food knowledge, and self-efficacy for native Canadian children. J Nutr. 2005;135(10):2392-8.

Kakekagumick KE, Hayward MN, Harris SB, Saksvig B, Gittelsohn J, Manokeesic G, Goodman S, Hanley AJ. Sandy lake health and diabetes project: a community-based intervention targeting type 2 diabetes and its risk factors in a first nations community. Front Endocrinol. 2013;4. https://doi.org/10.3389/fendo.2013.00170

**Starr County**

Brown SA, Hanis CL. Culturally competent diabetes education for Mexican Americans: the Starr County study. Diabetes Educ. 1999;25(2):226-36.

Brown SA, Garcia AA, Kouzekanani K, Hanis CL. Culturally competent diabetes self-management education for Mexican Americans. Diabetes Care. 2002;25(2):259-68.

**Yakima Valley**

Livaudais JC, Thompson B, Islas I, Ibarra G, Godina R, Coronado GD. Type 2 diabetes among rural Hispanics in Washington State: perspectives from community stakeholders. Health Promot Pract. 2010;11(4):589-99.

Duggan C, Carosso E, Mariscal N, Islas I, Ibarra G, Holte S, Copeland W, Linde S, Thompson B. Peer Reviewed: Diabetes Prevention in Hispanics: Report From a Randomized Controlled Trial. Prev Chronic Dis. 2014;11
